# Coin-cidence? Have cashless payments reduced the incidence of upper aerodigestive foreign body insertion? A study of UK Hospital Episode Statistics

**DOI:** 10.1308/rcsann.2024.0050

**Published:** 2024-06-05

**Authors:** A Jangan, E Watts, M Pankhania

**Affiliations:** ^1^ Walsall Healthcare NHS Trust, UK; ^2^ University Hospitals Birmingham NHS Foundation Trust, UK; ^3^ The Rotherham NHS Foundation Trust, UK

**Keywords:** Hospital Episode Statistics, Coin foreign body, Upper aerodigestive tract foreign body, Cashless payments, Contactless payments

## Abstract

**Objectives:**

Insertions of nasal and oral foreign bodies (FB) are common presentations in the emergency department, with coins frequently implicated among paediatric populations. Contactless payments were first introduced in the UK in 2007, and cash payments significantly declined from 2012. This study aims to explore the potential implications of increasing contactless payments on FB ingestion.

**Methods:**

UK Hospital Episode Statistics (HES) were reviewed between 2000 and 2022. All FB retrieval procedures involving the alimentary tract, respiratory tract and nasal cavity were included. Regression analysis was performed to assess trends in the incidence of FB ingestion before and following the transition to cashless payments in 2012.

**Results:**

Following the decline in cash payments in 2012, the frequency of alimentary tract FB removal procedures decreased significantly by 27.78 procedures per year (*p* < 0.001). Similarly, respiratory FB removal procedure decreased by 4.83 per year (*p* = 0.009) and nasal cavity FB removal procedures decreased by 52.82 per year (*p* < 0.001).

**Conclusions:**

This study suggests a statistically significant decline in the number of procedures for removal of FB performed in the UK from 2012. Although this relationship is multifactorial, our data suggest an association between the introduction of contactless payments and a reduction in the number of FB retrieval procedures from the of upper aerodigestive tract.

## Introduction

Upper aerodigestive tract (UADT) foreign bodies (FBs) are a common presentation to the emergency department, particularly among paediatric populations.^1^ In the past, coins have been established as one of the most commonly ingested FBs globally.^2–4^ Coins are implicated in more than 75% of swallowed FBs in children under the age of 6.^5^ A review of endoscopies for FBs shows that 66% of the ingested FBs were coins.^6^ Coins are frequently ingested because of their thin, round shape and easy accessibility. Ingestion carries potentially significant morbidity if associated with airway obstruction or perforation.^2–4,7^ Furthermore, a recent study showed that the cost of FB removal is approximately £2,880,148 per annum.^1^

**Figure 1:**
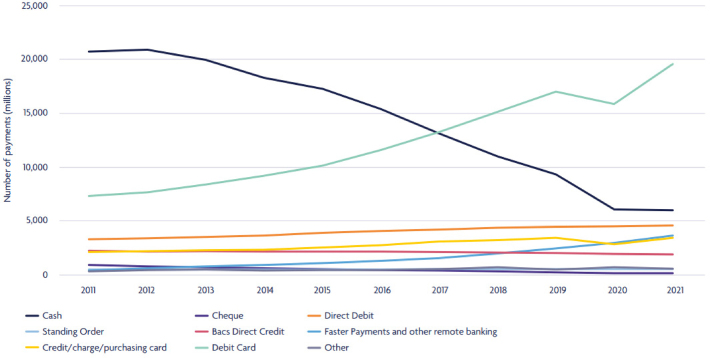
Methods of payment (millions) 2011 to 2021.^9^ Image adapted from: *UK payments markets summary*. U.K. Finance; 2022 [cited 15 April 2023]. Available from: https://www.ukfinance.org.uk/system/files/2022-08/UKF%20Payment%20Markets%20Summary%202022.pdf.

The first contactless card was released in the United Kingdom (UK) in 2007.^8^ The rapid adoption and adaptation of new technology (such as smart devices, online banking, smart wallets and contactless payments) have significantly impacted payment habits. UK Finance is a trade association of the UK banking and finance industry, representing more than 300 firms. It aims to analyse changing payment patterns and spending behaviour among the UK population. The 2022 UK Payment Markets Summary outlines changes to payment methods over the past decade.^9^ Figure 1, adapted from this report, highlights a gradual decline in the volume of cash payments since 2012.^9^ Cash payments have reduced from 62% of all payments made in the UK in 2006, to just 15% in 2021.^9^

Hospital Episode Statistics (HES) is a database produced by NHS Digital of all patient attendances across all the National Health Service (NHS) hospitals in the UK.^10^ All procedures are recorded using a four-character OPCS-4 code. This observational study aims to retrospectively determine the incidence of all procedures associated with UADT FB removal in children (<14 years) from April 2000 to April 2022 to determine the impact of contactless payments across this 22-year timeframe.

## Methods

HES data were accessed through the NHS Digital website, which detailed patient care activity for each fiscal year from April 2000 to April 2022.^10^ Codes (OPCS-4) from the procedures and interventions database (Table 1) were included in our study. Any procedures associated with FB removal distal to the lower oesophageal sphincter were excluded. Procedures were subdivided into three categories: removal of FBs from the respiratory tract, alimentary tract and nasal cavity (Table 1). The frequency of these procedures in 0–14-year-olds was recorded. This age group was predefined by the HES spreadsheet. The trend in procedures was assessed before and after 2012. According to the UK Payments Markets Survey, this is the year when cash payments began to decline, as seen in Figure 1.^9^ Therefore, this is considered the point of intervention.

**Table 1:** List of foreign body (FB) removal procedures included in the study.

	OPCS-4 code	Procedure
Nasal cavity FB removal procedures	E08.5	Removal of foreign body from cavity of nose
Respiratory tract FB removal procedures	E35.5	Endoscopic removal of foreign body from larynx
	E48.5	Fibreoptic endoscopic removal of foreign body from lower respiratory tract
	E50.5	Endoscopic removal of foreign body from lower respiratory tract using rigid bronchoscope
Alimentary tract FB removal procedures	E27.4	Removal of foreign body from pharynx
	F24.2	Removal of foreign body from tongue
	F32.2	Removal of foreign body from palate
	F36.4	Removal of foreign body from tonsil
	G13.2	Open removal of foreign body from oesophagus
	G15.1	Fibreoptic endoscopic removal of foreign body from oesophagus
	G18.1	Endoscopic removal of foreign body from oesophagus using rigid oesophagoscope

Coins are rarely inserted into the nasal cavity.^11–14^ We hypothesise that the incidence of this procedure would not be affected by an increased frequency of cashless payments, serving as a control procedure for this study. Coin FBs are more commonly found in the alimentary tract and therefore we hypothesise that these FB removal procedures would be impacted by cashless payments.

### Statistical analysis

Regression analysis was performed using R statistical software. Interrupted time series analysis was used to assess changes in the levels and trends in the frequency of FB removal procedures before and following the decline in cash payments in 2012 for all three groups of procedures. To aid analysis, starting in April 2000 each fiscal year was numbered from 1 to 22. Year 12 (April 2011 to April 2012) was considered the point of intervention. The regression coefficients describe the trend at three different points: before intervention, during intervention and post intervention.

## Results

Some 79,563 UADT FBs were removed across the 22-year study period, of which 57% (45,170) were removed from 0–14-year-olds.

### Removal of FB from the alimentary tract

**Figure 2:**
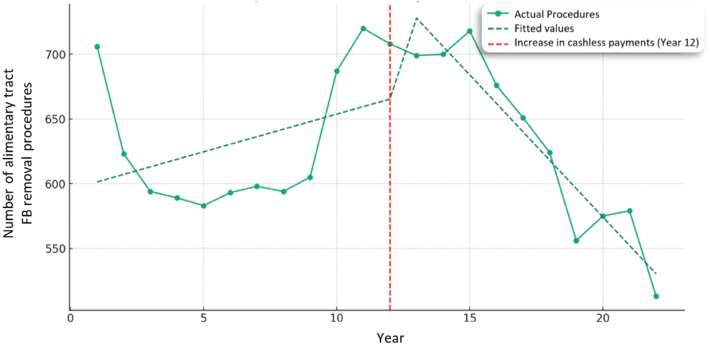
Interrupted time series analysis of alimentary tract foreign body removal procedures in 0–14-year-olds.

Regression coefficient calculations found no statistically significant increase in the number of procedures for FB removal from the alimentary tract between 2000 and 2012, with just 5.82 additional procedures per year (*p* = 0.120). In 2012, at the time of intervention, there was a nonsignificant increase of 56.76 procedures (*p* = 0.136). As cashless payments began to decline in 2012, we observe a statistically significant reduction in the frequency of FB removal procedures for the alimentary tract by 27.78 procedures per year (*p* < 0.001) (Figure 2).

### Removal of FB from the respiratory tract

**Figure 3:**
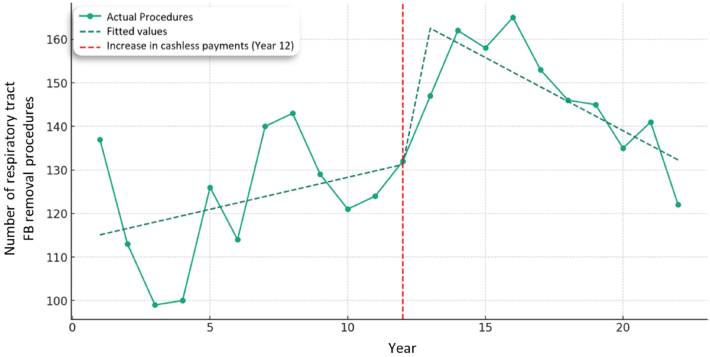
Interrupted time series analysis of respiratory tract foreign body removal procedures in 0–14-year-olds.

Regression analysis similarly found a non-statistically significant increase the number of patients requiring removal of FBs from the respiratory tract between 2000 and 2012 of just 1.47 procedures per year (*p* = 0.159). Interestingly, there was a significant increase in the number of FBs removed from the respiratory tract during 2012, with 29.80 more procedures this year (*p* = 0.009). Following the intervention year, 2012, there was a statistically significant reduction in the number of procedures by 4.83 fewer FB removal procedures per year (*p* = 0.009) (Figure 3).

### Removal of FB from the nasal cavity

**Figure 4:**
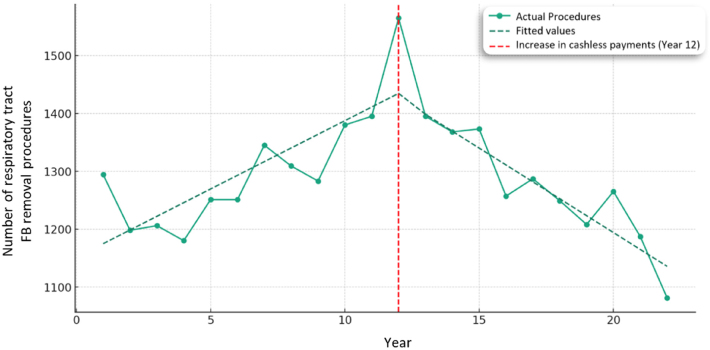
Interrupted time series analysis of nasal cavity foreign body removal in 0–14-year-olds.

A statistically significant increase of 23.62 additional nasal cavity FB removal procedures per year was found before the intervention in 2012 (*p* < 0.001). During the time of intervention (2012), there was a non-statistically significant reduction of 59.87 nasal cavity FB removal procedures per year (*p* = 0.236). Following 2012, this statistically significant reduction in nasal cavity FB removal procedures continued, reducing by 52.82 per year (*p* < 0.001) (Figure 4).

## Discussion

Overall, the numbers of FB removal procedures for the alimentary tract, respiratory tract and nasal cavity have reduced significantly over the past decade. A scoping literature review identified no other published studies investigating the implications of contactless or cashless payments on the inhalation or ingestion of coin FBs.

The observed trends might reflect changes in how data are collected, reported or recorded. An increase in awareness or improvements in healthcare access might lead to more reported cases initially, followed by a normalisation of data reporting. Additionally, there is likely an improvement in the reporting and coding of procedures, especially as technologies, practice and computer software within the NHS have evolved. In 2005 the Health Informatic Unit of the Royal College of Physicians aimed to improve the quality and accuracy of clinical coding through the iLab (Royal College of Physicians' information library) project.^15^

Many finance authorities report a decline in the use of cash payments and an uptake of cashless transactions between 2007 and 2012.^16^ Following the increase in cashless payments identified in 2012, we report a statistically significant reduction in the frequency of alimentary FB removal procedures. This study implies this change in consumer behaviour may have contributed to the multifactorial reduction in the incidence of FB removal procedures.

Conversely, our study observed a reduction in FB removal from the respiratory tract and nasal cavity. The most commonly implicated FBs within the nasal cavity include beads, pins, teeth, screws and food.^11–14^ Laryngo-tracheo-bronchial FBs are typically organic material such as nuts, peas and other foods.^17–20^ Because coins are less commonly found within the nasal cavity or upper airway, the concurrent reduction in laryngo-tracheo-bronchial and nasal FB removal procedures contradicts our original hypothesis. This suggests the overall reduction in FB ingestion and inhalation is likely multifactorial and may suggest a coincidental statistically significant decline in procedures as opposed to a causal association.

The significant decline in FB removal procedures identified across all three groups is likely multifactorial. Changes in public health policies, education programmes targeting children and parents, and shifts in population behaviours unrelated to payment methods may all play a part. One such contributing factor includes an education policy introduced by the Resuscitation Council in 2020 that sought to raise awareness of the management of acute Foreign Body Airway Obstruction.^21^ The availability and accessibility of these guidelines may have resulted in improved management of FB inhalation and ingestion in the community, thereby reducing the need for secondary care input or further procedural intervention. Suffocation because of FB inhalation is a leading cause of mortality in 0–3-year-olds with approximately 50,000 incidents per year in children aged 0–14.^22^ As a result, “The Susy Safe Project” was launched in 2008 to provide a risk analysis profile and to raise awareness of FB inhalation injuries to children.^22^ These public health initiatives may have played a significant role in the prevention of FB inhalation or ingestion.

It is important to recognise other environmental factors impacting the prevalence of FB ingestion or inhalation. The COVID-19 pandemic impacted social, economic and medical practices, leading to reduced hospital attendances and reduced operating, which could have influenced later reductions in FB procedures.^23^ In addition, as childcare centres closed and more parents worked from home, potential changes in child supervision levels may have occurred.^24^ This is reflected in multiple observational studies suggesting an increase in the incidence of FB ingestion during the COVID-19 pandemic.^25,26^ One such study show an increase in FBs located below the oesophagus because of delayed presentations during the COVID period. This resulted in a lesser need for endoscopic removal, which may have shifted the threshold for endoscopic surgery.^26^ In addition, there was an overall reduction in the number of admissions and surgical procedures. Furthermore, the Financial Conduct Authority reports the uptake of cashless payments was accelerated by the COVID pandemic to reduce the spread of disease.^27^

### Study limitations

A vital limitation of this study is that HES does not differentiate the type of FB ingested or inhaled. This study uses the generic HES and OPCS data as a surrogate marker for coin FBs. In addition, this study fails to account for the incidence of patients who swallow a coin and do not require operative intervention.

## Conclusion

Contactless and cashless payment systems have changed our spending habits, with many people no longer choosing to carry physical cash. Although this may have impacted the incidence of FB coin ingestion in children, we acknowledge such trends are likely multifactorial. The decline in FB removal procedures that is observed post 2012 across the other groups needs to be explored and assessed further to provide a more comprehensive understanding of the dynamics at play. We look forward to reviewing trends in the HES as contactless payments continue to predominate. To establish a more concrete causative relationship further data looking specifically at coin FBs need to be analysed.

## Data availability

The data that support the findings of this study are openly available on NHS Digital at https://digital.nhs.uk/data-and-information/publications/statistical/hospital-admitted-patient-care-activity.
